# Supplementary material for: Effectiveness of health partners coordination for COVID-19 pandemic response in Nepal
Source: PLoS One. 2024 Oct 16;19(10):e0308941. doi: 10.1371/journal.pone.0308941 (PMC11482675; doi:10.1371/journal.pone.0308941)
Supplement: S1 File — (PDF) [file pone.0308941.s001.pdf]

INDIVIDUAL MEETING'S DOCUMENT (AGENDA, PRESENTATIONS, MINUTES) REVIEW TOOL

Meeting number : ……………………

Meeting Date: …………………….

Meeting during:

- 1st wave (till 15 March 2021)
- 2nd wave (16 March 2021-7 Jan 2022)
- 3rd wave (8 Jan 2022-)

Number of participants: ………………………….

CONDUCTION

Which pillar were included in the agenda and what time duration (in minutes) was allocated?

- Country-level coordination, planning, and monitoring (Time allocated: ……… mins)
- Risk communication and community engagement (Time allocated: ……… mins)
- Surveillance, rapid response teams, and case investigation (Time allocated: ……… mins)
- Points of entry, international travel, and transport (Time allocated: ……… mins)
- National laboratories (Time allocated: ……… mins)
- Infection prevention and control (Time allocated: ……… mins)
- Case management (Time allocated: ……… mins)
- Operational support and logistics (Time allocated: ……… mins)
- Maintaining essential health services and systems (Time allocated: ……… mins)
- COVID-19 vaccines (Time allocated: ……… mins)

Was the purpose of the meeting met i.e., all the agenda items covered?

- Yes
- No

Were the action points of previous meeting followed up?

- Yes
- No

PROCESS

Were presentations from partners included in the meeting?

- Yes
- No

Time allocated for discussion: ………………………………… Mins

PARTICIPATION

Did all or most invitees attend? (actual present in the meeting/email sent is denominator>50%)

- Yes
- No

Was the participation as expected and as required? (addressed/recognized/acknowledged at least 50%)

- Yes
- No

FOLLOW-UP ACTIONS AND "NEXT STEPS"

Were the next steps and action items fully documented at the end of the meeting?

- Yes
- No

How were the action points/decisions framed? (completeness of at least 1 action point.)

- What
- What and Who
- What, Who and When
