# Supplementary material for: Effectiveness of health partners coordination for COVID-19 pandemic response in Nepal
Source: PLoS One. 2024 Oct 16;19(10):e0308941. doi: 10.1371/journal.pone.0308941 (PMC11482675; doi:10.1371/journal.pone.0308941)
Supplement: S2 File — (PDF) [file pone.0308941.s002.pdf]

Tool for Assessment of Health Coordination established for COVID-19 Pandemic in Nepal

1. General Information

1.1 Date: *

1.2 Do you participate in the cluster at? *

- o National level
- o Sub-national level
- o Both

1.3 Please provide the name of the sub-national level? *

- o Province 1
- o Madhesh Province
- o Bagmati Province
- o Gandaki Province
- o Lumbini Province
- o Karnali Province
- o Sudurpaschim Province

Country - Subnational Level

1.4 Do you work for? *

- o International NGO
- o National NGO
- o UN Organization
- o National Authority
- o Other
- o Donor

1.5 The organization you work for is a: *

- Lead
- Co-Lead
- Cluster member
- Cluster observer*
- Other
- Don't know

*Observers are not full members of the cluster but share information with the cluster and participate in cluster meetings

2.(A) Supporting service delivery

2.1Coordinating to ensure that service delivery is driven by the agreed strategic priorities

Please note: the questions in this section refer to all the meetings

2.1.1 Have regular coordination meetings been organized, as needed? *

- No meeting
- Meetings organised far less often than needed
- Meetings organised less often than needed
- Meetings organised almost as often as needed
- Meetings organised as often as needed
- Do not know

2.1.2 How was the attendance of your organization to cluster meetings? *

- Has not attended
- Has rarely attended
- Has sometimes attended
- Has often attended
- Has always attended
- Do not know

2.1.3 Has the staff attending the meetings had decision-making power and ability to follow-up on decisions made? *

- No decision-making power and ability to follow-up on decisions made
- No decision-making power but ability to follow-up on decisions made
- Limited decision-making power and ability to follow-up on decisions made
- Limited decision-making power but ability to follow-up on decisions made

- Full decision-making power and ability to follow-up on decisions made

2.1.4 Has the cluster created conditions for optimal participation of national and international stakeholders in meetings, e.g. location of meetings, language, methods? *

- Poor conditions
- Average conditions
- Good conditions
- Very good conditions
- Do not know

2.1.5 Have cluster meetings been useful in general for discussing needs, gaps and priorities in response? *

- Not useful
- Somewhat useful
- Mostly useful
- Very useful
- Do not know

2.1.6 Have useful strategic directions been agreed within the cluster? *

- No strategic directions agreed
- Strategic directions not useful
- Strategic directions somewhat useful
- Strategic directions mostly useful
- Strategic directions very useful
- Do not know

2.(B) Supporting service delivery

2.2 Developing mechanisms to eliminate duplication of service delivery

Please note: the questions in this section refer to all the meetings

2.2.1 Has your organization provided inputs into the mapping of partner geographic presence and programme activities, e.g. 3W and other similar mechanisms, as required? *

- No mapping
- Mapping done but no inputs from my organization
- Mapping done and inputs from my organization far less often than required
- Mapping done and inputs from my organization less often than required
- Mapping done and inputs from my organization almost as often as required
- Mapping done and inputs from my organization as often as required
- Do not know
- Not applicable for my organization (e.g. donors)

2.2.2 Has your organization been involved in the analysis of capacity and complementarity, i.e. gaps and overlaps, based on the mapping of partners geographic presence and programme activities? *

- No analysis
- My organization was not asked to participate
- My organization was asked to participate but has not contributed
- My organization was asked to participate, has contributed but contribution not adequately taken into account
- My organization was asked to participate, has contributed and contribution somewhat adequately taken into account
- My organization was asked to participate, has contributed and contribution adequately taken into account
- Do not know
- Not applicable for my organization (e.g. observers not engaging in this cluster activity)

2.2.3 Has the analysis of complementarity, i.e. gaps and overlaps, based on the mapping of

partner geographic presence and programme activities, been used by your organization for decision-making? *

- o Analysis not used for decision-making
- o Analysis seldom used for decision-making
- o Analysis sometimes used for decision-making
- o Analysis often used for decision-making
- o Analysis always used for decision-making
- o Do not know

2.2.4 Please enter below any further comments/clarifications you might have about the cluster's support to service delivery:

3.(A) Informing strategic decision-making of the HC/HCT for the humanitarian response

3.1 Needs assessment and gap analysis

Please note: the questions in this section refer to all the meetings

3.1.1 Has your organization used sectoral needs assessment tools and guidance agreed upon by cluster partners? *

- No agreed assessment tools and guidance
- Cluster agreed assessment tools and guidance not used by my organization
- Cluster agreed assessment tools and guidance seldom used by my organization
- Cluster agreed assessment tools and guidance sometimes used by my organization
- Cluster agreed assessment tools and guidance often used by my organization
- Cluster agreed assessment tools and guidance always used by my organization
- Do not know

3.1.2 Has your organization been involved in sectoral joint needs assessment and surveys? *

- No joint assessment or survey done
- Organization not involved in joint assessments and surveys
- Organization involved in few joint assessments and surveys
- Organization involved in some joint assessments and surveys
- Organization involved in most joint assessments and surveys
- Organization involved in all joint assessments and surveys
- Do not know
- Not applicable for my organization (e.g. observers not engaging in this cluster activity)

3.1.3 Has your organization shared with the cluster reports of surveys and assessments it has undertaken? *

- No surveys or assessments done
- No survey or assessment report shared
- Few survey and assessment reports shared
- Some survey and assessment reports shared
- Most survey and assessment reports shared
- All survey and assessment reports shared
- Do not know
- Not applicable for my organization (e.g. observers not engaging in this cluster activity)

3.(B) Informing strategic decision-making of the HC/HCT for the humanitarian response

3.2 Analysis to identify (emerging) gaps, obstacles, duplication and cross-cutting issues

Please note: the questions in this section refer to all the meetings

3.2.1 Have analyses of situations been done together with cluster partners? *

- o No
- o Yes
- o Do not know

3.2.2 To what extent have these analyses identified risks, needs, gaps, capacity and/or constraints to respond? *

	Not identified	Partially identified	Mostly identified	Fully identified	Do not know
Risks	O	O	O	O	O
Needs	O	O	O	O	O
Gaps in response	O	O	O	O	O
Capacity to response	O	O	O	O	O
Constraints to response	O	O	O	O	O

3.2.3 To what extent have cross cutting issues been considered in joint analyses? *

	Not considered	Partially considered	Mostly considered	Fully considered	Do not know
Age	O	O	O	O	O
Gender	O	O	O	O	O
Diversity (other than age and gender)	O	O	O	O	O
Human rights	O	O	O	O	O
Protection, including sexual and gender based violence	O	O	O	O	O
Environment	O	O	O	O	O
HIV/AIDS	O	O	O	O	O
Disability	O	O	O	O	O
Marginalized groups	O	O	O	O	O
Hard to reach areas	O	O	O	O	O

3.(C) Informing strategic decision-making of the HC/HCT for the humanitarian response

3.3 Prioritization grounded in response analysis

Please note: the questions of this section refer to all the meetings

3.3.1 Have joint analyses supported response planning and prioritization?

*

- Joint analyses done but not used to support response planning and prioritization
- Joint analyses sometimes used to support response planning and prioritization
- Joint analyses often used to support response planning and prioritization
- Joint analyses always used to support response planning and prioritization
- Do not know

3.3.2 Please enter below any further comments/clarifications you might have about the role of the cluster in informing strategic decision-making of the HC/HCT for the humanitarian response:

Please write your answer here:

4.(A) Planning and strategy development

4.1 Developing sectoral plans, objectives, indicators directly supporting HC/HCT strategic priorities

4.1.1 Has your organization been involved in the development of the sectoral strategic plan? *

- No sectoral strategic plan
- Organization not asked to participate
- Organization asked to participate but has not contributed
- Organization asked to participate, has contributed but contribution not adequately taken into account
- Organization asked to participate, has contributed and contribution somewhat adequately taken into account
- Organization asked to participate, has contributed and contribution adequately taken into account
- Do not know
- Not applicable for my organization (e.g. observers not engaging in this cluster activity)

4.1.2 Has the sectoral strategic plan guided response from your organization in the last 6 months? *

- Strategic plan but not shared with my organization
- Strategic plan shared but not used by my organization
- Strategic plan shared and sometimes used by my organization
- Strategic plan shared and often used by my organization
- Strategic plan shared and always used by my organization
- Do not know

4.(B) Planning and strategy development

4.2Application and adherence to existing standards and guidelines

Please note: the questions in this section refer to all the meetings

4.2.1 Have technical standards and guidance* been agreed by cluster partners and applied by your organization? *

- No technical standards and guidance agreed
- Technical standards and guidance agreed but not used
- Technical standards and guidance agreed and sometimes used
- Technical standards and guidance agreed and often used
- Technical standards and guidance agreed and always used
- Do not know

*Technical standards and guidance could be issued either by specific technical working groups or through discussion at cluster meetings

4.(C) Planning and strategy development

4.3 Clarifying funding needs, prioritization and cluster contributions to HC funding considerations (e.g. MoHP Rapid Action Plan, UN Nepal Country Preparedness & Response Plan)

Please note: the questions in this section refer to all the meetings

4.3.1 Have prioritization of proposals against the strategic plan been jointly determined with cluster partners based on agreed transparent criteria? *

- No agreed transparent criteria and prioritization not jointly determined with partners
- No agreed transparent criteria but prioritization jointly determined with partners
- Agreed transparent criteria but prioritization not jointly determined with partners
- Agreed transparent criteria and prioritization somewhat determined jointly with partners
- Agreed transparent criteria and prioritization fully determined jointly with partners
- Do not know

4.3.2 To which extent has the prioritization of proposals against the strategic plan reflected the interest of cluster partners? *

- No prioritization of proposals
- Prioritization of proposals not reflecting the interests of partners
- Prioritization of proposals reflecting the interests of few partners
- Prioritization of proposals reflecting the interests of most partners
- Prioritization of proposals reflecting the interests of all partners
- Do not know

4.3.3 How often has the cluster coordinator reported on funding status of the cluster against the needs*? *

- o Never
- o Far less often than needed
- o Less often than needed
- o Almost as often as needed
- o As often as needed
- o Do not know

*e.g. tracking of funds received against CAP and other appeals and proposals

4.3.4 Please enter below any further comments/clarifications you might have about cluster's role in planning and strategy development:

5.(A) Advocacy

5.1 Identifying advocacy concerns to contribute to HC and HCT messaging and action

Please note: the questions in this section refer to all the meetings

5.1.1 Have issues requiring advocacy been identified and discussed together with your organization? *

- o No issues discussed
- o Issues discussed but my organization was not asked to participate
- o Issues discussed, my organization was asked to participate but did not contribute
- o Issues discussed, my organization was asked to participate, has contributed but views not adequately taken into account
- o Issues discussed, my organization was asked to participate, has contributed and views adequately taken into account
- o Do not know
- o Not applicable for my organization (e.g. observers not engaging in this cluster activity)

5.(B) Advocacy

5.2 Undertaking advocacy activities on behalf of cluster participants and the affected population

Please note: the questions in this section refer to all the meetings

5.2.1 Has your organization participated in cluster advocacy activities? *

- No cluster advocacy activity
- Cluster advocacy activities but my organization was not asked to participate
- Cluster advocacy activities, my organization was asked to participate but did not
- Cluster advocacy activities, my organization was asked to participate and did participate in some activities
- Cluster advocacy activities, my organization was asked to participate and did participate in most activities
- Do not know
- Not applicable for my organization (e.g. observers not engaging in this cluster activity)

5.2.2 Please enter below any further comments/clarifications you might have about cluster's role in advocacy activities:

6. Monitoring and reporting on the implementation of the cluster strategy and results recommending corrective action where necessary

Please note: the questions in this section refer to all the meetings

6.1 Have changes in needs, risks and gaps highlighted in cluster bulletins and other reports been used for decision making by your organization? *

- Changes in needs, risks and gaps not highlighted in any bulletins or reports
- Changes in needs, risks and gaps highlighted but not used for decision-making
- Changes in needs, risks and gaps highlighted and sometimes used for decision-making
- Changes in needs, risks and gaps highlighted and often used for decision-making
- Changes in needs, risks and gaps highlighted and always used for decision-making
- Do not know

6.2 Have partners used programme monitoring and reporting formats agreed upon by cluster partners? *

- No standards agreed for monitoring and reporting
- Standards agreed but not reported on regularly by my organization
- Standards agreed and reported on somewhat regularly by my organization
- Standards agreed and reported on regularly by my organization
- Standards agreed and reported on very regularly by my organization
- Do not know

6.3 To what extent have the monitoring and response of your cluster taken into account the distinct needs, contributions and capacities of women, girls, men and boys, when applicable*? *

- Not applicable due to the nature of activities
- Not considered
- Marginally considered

- Partially considered
- Mostly considered
- Fully considered
- Do not know

* Not applicable if cluster activities do not imply a direct contact with affected populations, and do not directly affect or determine the selection or use of resources, goods or services accessed by affected populations

6.4 Please enter below any further comments/clarifications you might have about the monitoring and reporting of the implementation of the cluster strategy and results:

7. Preparedness for recurrent disasters whenever feasible and relevant

Please note: the questions in this section refer to all the meetings

7.1 Has your organization been involved in the development and/or updates of the preparedness plan (multisectoral where appropriate) for all risks/hazards? *

- Preparedness plan not done/updated
- Preparedness plan done/updated but my organization was not asked to participate
- Preparedness plan done/updated, my organization was asked to participate, but did not
- Preparedness plan done/updated, my organization was asked to participate but has inadequately contributed
- Preparedness plan done/updated, my organization was asked to participate and has adequately contributed

- o Do not know
- o Not applicable for my organization (e.g. observers not engaging in this cluster activity)

7.2 Has your organization committed staff and/or resources to be mobilized when preparedness plans will be activated? *

- o No staff or resources committed
- o Limited staff and/or resources committed
- o Adequate staff and/or resources committed
- o Do not know
- o Not applicable for my organization (e.g. observers not engaging in this cluster activity)

7.3 Please enter below any further comments/clarifications you might have about preparedness:

8. Accountability to affected populations

Please note: the questions in this section refer to all the meetings

8.1 Have mechanisms - agreed upon by cluster partners - to consult and involve affected populations in decision-making*, been used by your organization when possible? *

- No mechanism agreed
- Mechanisms agreed but not used
- Mechanisms agreed and seldom used
- Mechanisms agreed and sometimes used
- Mechanisms agreed and often used
- Mechanisms agreed and always used
- Do not know

*in line with the IASC principals commitments on accountability to affected populations (CAAP)

8.2 Have mechanisms - agreed upon by cluster partners - to receive, investigate and act upon complaints on the assistance received*, been used by your organization when possible? *

- No mechanism agreed
- Mechanisms agreed but not used
- Mechanisms agreed and seldom used
- Mechanisms agreed and sometimes used
- Mechanisms agreed and often used
- Mechanisms agreed and always used
- Do not know

*in line with the IASC principals commitments on accountability to affected populations (CAAP)

8.3 Please enter below any further comments/clarifications you might have about accountability to affected populations and any information on how feedback systems are performing:

