# Supplementary material for: Effectiveness of health partners coordination for COVID-19 pandemic response in Nepal
Source: PLoS One. 2024 Oct 16;19(10):e0308941. doi: 10.1371/journal.pone.0308941 (PMC11482675; doi:10.1371/journal.pone.0308941)
Supplement: S1 Table — (PDF) [file pone.0308941.s003.pdf]

S1 Table. Interview Guide for Focus Group Discussion.

THEME	LEAD QUESTIONS	PROBING QUESTIONS
**CONDUCTION**	Did the meetings meet the purpose?	• Were all the pillars for COVID-19 response adequately addressed?<br>• Did you feel any of the issues left out/less addressed? If yes, what and how do you suggest its inclusion?
**PROCESS**	Was the correct mechanism and venue used?	• What do you think would have been better physical meeting, phone conference, video conference? Why?
	Could better results have been achieved through a different meeting mechanism?	• Why/why not?
	Were any technical problems experienced?	• Could you explain?<br>• Any suggestions to minimize the issue?
	Were any logistical problems experienced?	• Could you explain?<br>• Any suggestions to minimize the issue?
**PARTICIPATION**	Did the meetings have right mix of attendees and participants?	• Did you feel any of the organization /health partners left out?
	Were participation roles and responsibilities communicated and clarified prior to the start of the meeting?	• Was the mechanism appropriate?<br>• Are any changes required for communication?
	Was the discussion properly controlled and managed?	• Who and How?<br>• Were certain individuals allowed to dominate the discussion to the detriment of others? If yes, could you explain more?<br>• Did the meetings have positive or negative tone? Could you explain?
**NEXT STEPS**	Were there clear actions points and their follow up?	• Who was involved and what procedures were followed to ensure that assignments and next steps were properly executed and completed?
