# Supplementary material for: Effectiveness of health partners coordination for COVID-19 pandemic response in Nepal
Source: PLoS One. 2024 Oct 16;19(10):e0308941. doi: 10.1371/journal.pone.0308941 (PMC11482675; doi:10.1371/journal.pone.0308941)
Supplement: Attachment — Submitted filename: PONE-D-24-09019_reviewer1.pdf [file pone.0308941.s005.pdf]

PLOS ONE

Effectiveness of Health Partners Coordination for COVID-19 pandemic response in Nepal

--Manuscript Draft--

**Manuscript Number:**	PONE-D-24-09019
**Article Type:**	Research Article
**Full Title:**	Effectiveness of Health Partners Coordination for COVID-19 pandemic response in Nepal
**Short Title:**	Effectiveness of Health Partners Coordination for COVID-19 pandemic response in Nepal
**Corresponding Author:**	Gaurav Devkota, MBBS, MPH<br>World Health Organization Country Office for Nepal<br>Lalitpur, NEPAL
**Keywords:**	Coordination, COVID-19, Health Partner, Qualitative, Survey, Tools
**Abstract:**	Background: Nepal established the health partner coordination for COVID-19 response based on national and international plans and framework to support information-sharing for decision-making and course correction. The objective of this study was to assess the performance of the Health Partner Coordination established for COVID-19 response in Nepal by adopting coordination best practices and tools from the international humanitarian system.<br>Methods: An analysis of secondary data from unpublished documents collected on health partner coordination for COVID-19 response in Nepal was conducted from April 2023 to May 2023. Information available were from the review of the documents of the 72 health partner coordination meetings conducted for COVID-19 response using a self-developed document review tool, responses of 26 health partners on a survey tool adapted from the cluster coordination performance monitoring tool from the Global Health Cluster, and transcript of Focused Group Discussion among selected seven health partners. Descriptive analysis of quantitative information and thematic analysis with predefined themes of qualitative information were performed using MS Excel and MS Word respectively. A written approval from the Ministry of Health and Population and an ethical clearance from Nepal Health Research Council was obtained before the conduction of the study.<br>Results: More than three-fifth of the meetings showed good results in conduction, process, participation, and documentation of action points with improvement required for follow-up. Majority of the activities of the health partner coordination were good (>75.0%) or satisfactory (50.1%-75.0%) except for prioritization based on analyses. Partners admired good practices of health partner coordination, pointed out some issues, and provided recommendations.<br>Conclusion: COVID-19 has highlighted the importance of effective coordination of health sector for response to a pandemic. Continuation of this practice after addressing the areas for improvement will contribute to health preparedness and readiness for future disasters or public health emergencies.
**Order of Authors:**	Gaurav Devkota, MPH
	Sangeeta Kaushal Mishra, MPH
	Samir Kumar Adhikari, MPH
	Pavan Kumar Sah, MDGP
	Allison Eugenio Gocotano, MIPH
	Subash Neupane, MCMS
	Barsha Thapa, BN
**Additional Information:**	
**Question**	**Response**

Powered by Editorial Manager® and ProduXion Manager® from Aries Systems Corporation

**Financial Disclosure**<br><br>Enter a financial disclosure statement that describes the sources of funding for the work included in this submission. Review the submission guidelines for detailed requirements. View published research articles from *PLOS ONE* for specific examples.<br><br>This statement is required for submission and **will appear in the published article** if the submission is accepted. Please make sure it is accurate.<br><br>**Funded studies**<br>Enter a statement with the following details:<br>• Initials of the authors who received each award<br>• Grant numbers awarded to each author<br>• The full name of each funder<br>• URL of each funder website<br>• Did the sponsors or funders play any role in the study design, data collection and analysis, decision to publish, or preparation of the manuscript?<br><br>Did you receive funding for this work?	The author(s) received no specific funding for this work.
**Competing Interests**<br><br>Use the instructions below to enter a competing interest statement for this submission. On behalf of all authors, disclose any competing interests that could be perceived to bias this work—acknowledging all financial support and any other relevant financial or non-financial competing interests.<br><br>This statement is required for submission and **will appear in the published article** if the submission is accepted. Please make sure it is accurate and that any funding sources listed in your Funding Information later in the submission form are also declared in your Financial Disclosure statement.	The authors have declared that no competing interests exist.

View published research articles from *PLOS ONE* for specific examples.<br><br>**NO authors have competing interests**<br><br>Enter: *The authors have declared that no competing interests exist.*<br><br>**Authors with competing interests**<br><br>Enter competing interest details beginning with this statement:<br><br>*I have read the journal's policy and the authors of this manuscript have the following competing interests: [insert competing interests here]*<br><br>* typeset	
**Ethics Statement**<br><br>Enter an ethics statement for this submission. This statement is required if the study involved:<br><br>• Human participants<br>• Human specimens or tissue<br>• Vertebrate animals or cephalopods<br>• Vertebrate embryos or tissues<br>• Field research<br><br>Write "N/A" if the submission does not require an ethics statement.<br><br>General guidance is provided below. Consult the submission guidelines for detailed instructions. **Make sure that all information entered here is included in the Methods section of the manuscript.**	N/A

**Format for specific study types**<br><br>**Human Subject Research (involving human participants and/or tissue)**<br>• Give the name of the institutional review board or ethics committee that approved the study<br>• Include the approval number and/or a statement indicating approval of this research<br>• Indicate the form of consent obtained (written/oral) or the reason that consent was not obtained (e.g. the data were analyzed anonymously)<br><br>**Animal Research (involving vertebrate animals, embryos or tissues)**<br>• Provide the name of the Institutional Animal Care and Use Committee (IACUC) or other relevant ethics board that reviewed the study protocol, and indicate whether they approved this research or granted a formal waiver of ethical approval<br>• Include an approval number if one was obtained<br>• If the study involved *non-human primates*, add *additional details* about animal welfare and steps taken to ameliorate suffering<br>• If anesthesia, euthanasia, or any kind of animal sacrifice is part of the study, include briefly which substances and/or methods were applied<br><br>**Field Research**<br><br>Include the following details if this study involves the collection of plant, animal, or other materials from a natural setting:<br>• Field permit number<br>• Name of the institution or relevant body that granted permission	
**Data Availability**<br><br>Authors are required to make all data underlying the findings described fully available, without restriction, and from the time of publication. PLOS allows rare exceptions to address legal and ethical concerns. See the PLOS Data Policy and FAQ for detailed information.	No - some restrictions will apply

A Data Availability Statement describing where the data can be found is required at submission. Your answers to this question constitute the Data Availability Statement and **will be published in the article**, if accepted.<br><br>**Important:** Stating 'data available on request from the author' is not sufficient. If your data are only available upon request, select 'No' for the first question and explain your exceptional situation in the text box.<br><br>Do the authors confirm that all data underlying the findings described in their manuscript are fully available without restriction?	
**Describe where the data may be found in full sentences. If you are copying our sample text, replace any instances of XXX with the appropriate details.**<br><br>• If the data are **held or will be held in a public repository**, include URLs, accession numbers or DOIs. If this information will only be available after acceptance, indicate this by ticking the box below. For example: *All XXX files are available from the XXX database (accession number(s) XXX, XXX.*).<br>• If the data are all contained **within the manuscript and/or Supporting Information files**, enter the following: *All relevant data are within the manuscript and its Supporting Information files.*<br>• If neither of these applies but you are able to provide **details of access elsewhere**, with or without limitations, please do so. For example:<br><br>*Data cannot be shared publicly because of [XXX]. Data are available from the XXX Institutional Data Access / Ethics Committee (contact via XXX) for researchers who meet the criteria for access to confidential data.*<br><br>*The data underlying the results presented in the study are available from (include the name of the third party*	Deidentified data are available from the Health Emergency Operation Center (HEOC), Ministry of Health and Population, Nepal upon request to heocmohp@gmail.com. All the authors have full access to the deidentified data through HEOC and take responsibility for the accuracy of the study.

and contact information or URL).<br>• This text is appropriate if the data are owned by a third party and authors do not have permission to share the data.<br><br>* typeset	
Additional data availability information:	

Manuscript Click here to access/download;Manuscript;rev2_Manuscript_Health partner

Effectiveness of Health Partners Coordination for COVID-19 pandemic response in Nepal

Sangeeta Kaushal Mishra[1], Samir Kumar Adhikari[1], Pavan Kumar Sah[1], Allison Eugenio Gocotano[2], Subash Neupane[2], Barsha Thapa[2], Gaurav Devkota[2*]

[1] Ministry of Health and Population, Government of Nepal, Kathmandu, Nepal

[2] World Health Organization Country Office for Nepal, Lalitpur, Nepal

* Corresponding author: Gaurav Devkota

Email: devkotag@who.int; gdevkota74@gmail.com

Phone number: +9779803513520, +9779867154474

Dr Sangeeta Kaushal Mishra: https://orcid.org/0000-0003-4419-6386

Dr Samir Kumar Adhikari: https://orcid.org/0000-0002-3226-4428

Dr Pavan Kumar Sah: https://orcid.org/0009-0002-3251-9395

Dr Allison Eugenio Gocotano: https://orcid.org/0009-0002-0448-9889

Dr Subash Neupane: https://orcid.org/0000-0002-4131-6381

Barsha Thapa: https://orcid.org/0009-0004-2855-634X

Dr Gaurav Devkota: https://orcid.org/0000-0003-0754-2534

Abstract

Background: Nepal established the health partner coordination for COVID-19 response based on national and international plans and framework to support information-sharing for decision-making and course correction. The objective of this study was to assess the performance of the Health Partner Coordination established for COVID-19 response in Nepal by adopting coordination best practices and tools from the international humanitarian system.

Methods: An analysis of secondary data from unpublished documents collected on health partner coordination for COVID-19 response in Nepal was conducted from April 2023 to May 2023. Information available were from the review of the 72 health partner coordination meetings conducted for COVID-19 response using a self-developed document review tool, responses of 26 health partners on a survey tool adapted from the cluster coordination performance monitoring tool from the Global Health Cluster, and transcript of Focused Group Discussion among selected seven health partners. Descriptive analysis of quantitative information and thematic analysis with predefined themes of qualitative information were performed using MS Excel and MS Word respectively. A written approval from the Ministry of Health and Population and an ethical clearance from Nepal Health Research Council was obtained before the conduction of the study.

Results: More than three-fifth of the meetings showed good results in conduction, process, participation, and documentation of action points with improvement required for follow-up. Majority of the activities of the health partner coordination were good (>75·0%) or satisfactory (50·1%-75·0%) except for prioritization based on analyses. Partners admired good practices of health partner coordination, pointed out some issues, and provided recommendations.

Conclusion: COVID-19 has highlighted the importance of effective coordination of health sector for response to a pandemic. Continuation of this practice after addressing the areas for improvement will contribute to health preparedness and readiness for future disasters or public health emergencies.

Keywords: Coordination, COVID-19, Health Partner, Qualitative, Survey, Tools

Introduction

World Health Organization (WHO) declared COVID-19 as Public Health Emergency of International Concern on 30 January 2020 and later as a pandemic on 11 March 2020. Nepal confirmed its first COVID-19 case on 23 January 2020.[1] As stated in United Nations General Assembly resolution 46/182 of 1991 (paragraphs 4–6) on national authorities having the primary responsibility for taking care of the victims of the natural disasters and other emergencies occurring in their territories,[2] national authorities' focus was on protecting health and saving lives of people while minimizing the disruptive effect of the pandemic on the economy and society which called for multisectoral coordination. This is reiterated in the purpose and scope of the International Health Regulations (IHR) which are *"to prevent, protect against, control and provide a public health response to the international spread of disease in ways that are commensurate with and restricted to public health risks, and which avoid unnecessary interference with international traffic and trade*."[3]

Coordination can be defined as "the attitudes, behaviors, and outcomes of joint determination of common (interorganizational relationship) goals".[4] Coordination during humanitarian response to a disaster or public health emergency is not as straightforward as a set of specific behaviors that call for urgency to maximize the benefits and minimize inefficiencies, but is the first step towards an effective, efficient, and sustainable response that builds the foundations of short- and long-term recovery.[5] One of the models for response coordination to disasters and public health emergencies is the cluster approach. Clusters are groups of humanitarian organizations (UN and non-UN) working in the main sectors of response, e.g., health, nutrition, shelter, etc. with systematic and standardized procedures appropriate to each sector's responsibilities and with systems in place for interactions between and among sectors.[6] The Inter-Agency Standing Committee (IASC) endorsed the Cluster Coordination Performance

Monitoring (CCPM) process and final format in August 2012 as a standardized means to monitor the performance of country-level cluster coordination.[7]

The WHO proposed ten pillars included in a COVID‑19 Strategic Preparedness and Response Plan. The first pillar being the Coordination, Planning, Financing, and Monitoring pillar. The coordination part is envisioned to play a role in ensuring coherence and operational alignment of the response at national and subnational levels impacting all other pillars. Moreover, the coordination pillar is expected to serve as the platform for ongoing decision-making and course correction based on available information.[8] In Nepal, the humanitarian cluster approach was adapted in the National Disaster Response Framework (2013) where it specifies in section 5 on national and international assistance coordination framework, that for Health, the lead government agency is the Ministry of Health and Population (MoHP) with WHO as co-lead agency.[9] The same approach was implemented during the COVID-19 pandemic for health partner coordination wherein Health Coordination Division (HCD) of the MoHP acted as lead and WHO Nepal as co-lead. National and subnational coordination was conducted through, and with information management and secretariat support by, the Health Emergency Operation Center (HEOC) at national and provincial levels. HCD chaired national-level coordination meetings among health partners.

The first coordination meeting among health partners was held on 9 April 2020,[1] continuing throughout the different COVID-19 infection transmission waves in Nepal, attributable to the delta (estimated period covered from March through December 2021) and omicron (estimated period from December 2021 to 2022) variants. A total of 72 meetings were conducted till mid-February 2022. The frequency of the meetings varied as per the COVID-19 situation, that is from weekly, two-weekly, monthly, to as required. Those meetings were chaired by HCD and co-chaired by WHO. The meetings, which acted as the gap-identifying platform for health

stakeholders to act upon, included information-sharing presentations from government and partners to foster a common understanding of the shared operational landscape, and discussions on the next steps and agreeing on priority action points. Due to the adherence to the prevailing public health and social measures, all these meetings were conducted virtually with participation of organizations based at both national and subnational levels. Decisions and action points were shared with all the partners and followed-up through the responsible focal points to demonstrate transparency and shared accountability.

HCD led the coordination of all the health partners for COVID-19 response following the provisions from the National Disaster Response Framework. These coordination meetings continued throughout different infection transmission waves of COVID-19 in Nepal, which provided a platform to share information and experiences, discuss challenges, identify gaps, and support coordinated actions. As the COVID-19 cases were reducing, HCD decided to assess the effectiveness of the health partner coordination with WHO's support to identify improvements of such coordination meetings in future similar scenarios and explore the usefulness of the coordination platform.

Methods

This study was conducted from April 2023 to May 2023 that included collection, review, and analysis of secondary information on health partner coordination. This information was collected from three means and were available with the MoHP. First was a collection, review, and analysis of unpublished documents and files of the meetings conducted by HCD with health partners for COVID-19 response from March 2020 to February 2022. A total of 72 meetings were documented. Individual meeting's documents including agenda, meeting minutes, and presentations were assessed according to four different themes (conduction, process, participation, and follow-up actions/next steps) through the document review tool (S1 File)

developed based on the meeting evaluation checklist[10] and post-meeting assessment steps[11] available online.

Second was analysis of the information available through a survey tool modified from the cluster coordination performance monitoring tool from the Global Health Cluster (S2 File) which includes standard questions across the six core functions of the health cluster to: (1) support to service delivery, (2) inform strategic decision-making, (3) plan and develop strategy, (4) monitor and evaluate performance, (5) build national capacity in preparedness and contingency planning, and (6) undertake advocacy, along with questions related to accountability to affected populations.[12] The modified tool was pretested among the Provincial Health Emergency Operation Centers before being administered to the health partners. While the survey tool was disseminated to focal points of 82 organizations that were within the health partner coordination during the time of tool administration, responses were available from only 26 health partners (18 International Non-Governmental Organizations (INGOs), three United Nations (UN) Organizations, one Donor, and four Others) within the allocated 15 days (30 May 2022 to 13 June 2022) to the organizations to response after dissemination of the tool.

Third was analysis of the information available as transcript of a Focus Group Discussion (FGD) conducted by HCD among focal person from two UN agencies, two donors, two INGOs and one NGO. These stakeholders were selected based on their participation in more than 50% of the last 20 health coordination meetings conducted for COVID-19 response with an available attendance list. The FGD was conducted using an interview guideline (S3 File) with the questions directed to discuss on the major themes as conduction, process, participation, and way forward related to the coordination for COVID-19 response. The available transcript was the English translation of the recorded FGD which was conducted in Nepali.

A written approval from the Ministry of Health and Population was obtained to collect and analyze these unpublished documents and files. Ethical clearance from Nepal Health Research Council (8/2023 P) was obtained before the conduction of this analysis. As the study involved collection and analysis of secondary information, no human subjects were involved in the study. However, deidentified data were used for analysis and codes were provided to maintain anonymity of the person who had provided information initially/primarily.

Descriptive analysis was performed using MS Excel on the data that were available from the document review tool and the available responses on the survey tool. An overall performance score of greater than 75·0% was defined to be good, 50·1%-75·0% as satisfactory, 25·1%-50·0% as unsatisfactory, and less than or equal to 25·0% as weak for activities under each of the six core functions, including accountability to the affected population in the survey tool.[7] Thematic analysis was performed in MS Word on the available transcript of the FGD with the previously mentioned pre-identified themes (conduction, process, participation, and follow-up actions/next steps).

Anonymity of personnel and organization was maintained during analysis of data and information and dissemination of the results. Codes were provided to the participants of the Focus Group Discussion and these codes were used during analysis. Privacy of all the data and information was maintained in password protected data repository of Health Emergency Operation Center, Ministry of Health and Population and will be maintained for a period of ten years.

Results

Health Coordination Meetings' Document Review

There were 55 individual participants on average in each meeting. On average, 43·5 minutes were spent on presentations and 11·5 minutes for discussions in a typical one-hour meeting. Country level coordination, planning and monitoring pillar had the longest time allocated in a typical meeting (average of 17·2 minutes), followed by Surveillance, rapid response teams, and case investigation pillar (average of 6·3 minutes). Figure 1 depicts that the frequency of the meetings was high at the early period of the pandemic and during surge of cases.

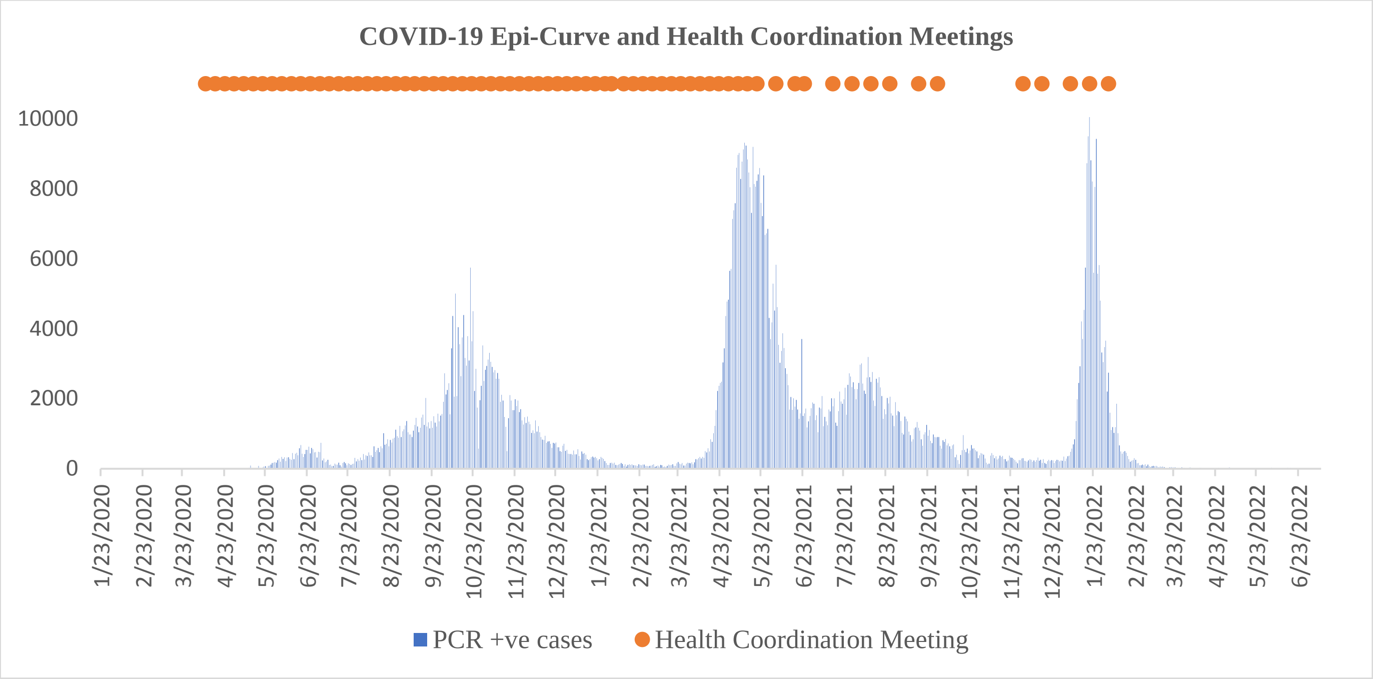

Figure 1: COVID-19 epidemic curve of Nepal and health coordination meetings, Jan 2020 to Jun 2022

Table 1 provides the analysis of the 72 health coordination meeting documents using the document review tool. 80·6% of the meetings covered all the agenda items while only 66·7% of the meetings followed up action points from the preceding meeting. Partners presented in 63·9% of the meetings with at least half of the invitees present in 73·6% of the meetings and at least half of the concerns addressed/recognized/acknowledged in 66·7% of the meetings. Finally, looking at follow-up actions and next steps, these were fully documented at 100·0% of meetings

while there is room for improvement in action point completion i.e., "who will be doing what activities and when" which was only documented in 22·2% of the meetings conducted.

Table 1: Analysis of the Health Coordination meeting documents

Themes	Questions		Number	Percentage
Conduction	Was the purpose of the meeting met i.e., all the agenda items covered?	No	14	19·4
		Yes	58	80·6
	Were the action points of previous meeting followed up?	No	24	33·3
		Yes	48	66·7
Process	Were presentations from partners included in the meeting?	No	26	36·1
		Yes	46	63·9
Participation	Did all or most (more than 50%) invitees attend?	No	19	26·4
		Yes	53	73·6
	Was the participation as expected and as required? (addressed/recognized/acknowledged at least 50% queries)	No	24	33·3
		Yes	48	66·7
Follow-up actions and next steps	Were the next steps and action items fully documented at the end of the meeting?	No	0	0·0
		Yes	72	100·0
	Were at least one action point complete (i.e., include who does what and when)?	No	56	77·8
		Yes	16	22·2

Performance monitoring of health partner coordination through survey

Table 2 depicts the analysis of the responses in the survey tool which shows *'Satisfactory'* results, i.e., 50·0%-75·0% score, in majority of the country health cluster core functions. The survey was administered from 30 May 2022 to 13 June 2022 by email to the primary focal point of each of the 82 organizations who participated in the health coordination meetings, and with response received only from 26 (31·7%) organizations. While the response rate is low, the organizations which responded had participation rate to the meetings of more than 50·0% whereas the organizations which did not respond had participation rate to the meetings of less than 50·0% (out of the 20 meetings with information available on participants' details).

Two areas yielded good results, i.e., a score of more than 75·0% score. First is the "Coordinating to ensure that service delivery is driven by the agreed strategic priorities" of the supporting service delivery function which covers activities related to coordination meetings like regularity, participation, partners' decision-making power and ability to follow-up decision made, usefulness in discussing needs, gaps, and priorities, and agreement on strategic direction. Second

is the "Application and adherence to existing standards and guidelines" of the planning and strategy development function which covers the activities like agreement on the technical standards and guidance by cluster members and its application by the responding organization.

“Prioritization grounded in response analysis” of the informing strategic decision-making function yielded *‘Unsatisfactory’* result, i.e., 25·0%-50·0% score. This covers the activities on the response planning and prioritization, supported by joint analyses.

Table 2: Analysis of the responses in the adapted Cluster Coordination Performance Monitoring tool

**1**	**Supporting service delivery**	
1.1	Coordinating to ensure that service delivery is driven by the agreed strategic priorities	*Good*
1.2	Developing mechanisms to eliminate duplication of service delivery	*Satisfactory*
**2**	**Informing strategic decision-making of the Humanitarian Coordinator (HC)/ Humanitarian Country Team (HCT) for the humanitarian response**	
2.1	Needs assessment and gap analysis	*Satisfactory*
2.2	Analysis to identify (emerging) gaps, obstacles, duplication, and cross-cutting issues	*Satisfactory*
2.3	Prioritization grounded in response analysis	*Unsatisfactory*
**3**	**Planning and strategy development**	
3.1	Developing sectoral plans, objectives, indicators directly supporting HC/HCT strategic priorities	*Satisfactory*
3.2	Application and adherence to existing standards and guidelines	*Good*
3.3	Clarifying funding needs, prioritization, and cluster contributions to HC funding considerations	*Satisfactory*
**4**	**Advocacy**	
4.1	Identifying advocacy concerns to contribute to HC and HCT messaging and action	*Satisfactory*
4.2	Undertaking advocacy activities on behalf of cluster participants and the affected population	*Satisfactory*
**5**	**Monitoring and reporting on the implementation of the cluster strategy and results**	*Satisfactory*
**6**	**Preparedness for recurrent disasters whenever feasible and relevant**	*Satisfactory*
**7**	**Accountability to affected populations**	*Satisfactory*

Thematic analysis of the Focus Group Discussion

Theme 1: Conduction

Participants responded to the HCD’s initiation of the health coordination meeting as a sign of good leadership and deemed them relevant in the context of COVID-19 pandemic response. Also, the fixed day and time for the meeting and the sharing of relevant documents were highly applauded.

"…the excitement, information sharing, has helped activate cluster rapidly. The leadership was appreciative…Next, fixed date and time was set, this somehow helped in time management and fixed schedule was maintained throughout the period... Reference documents, guidelines were shared time and again during and after the meeting" - Participant F

"In my opinion, the information flow was relevant. Especially, during the second wave it worked as a platform to obtain relevant information through one door mechanism." - Participant E

Participants also said that the health coordination meetings were the best platform to provide direction for COVID-19 management as it supported coordinated response. One of the participants mentioned that the meetings were limited to information sharing only.

"Umm... I want to reiterate the same thing. Like, if we look back at the situation during pandemic, the meeting worked as a platform in streamlining the necessary activities to be done. It provided us with direction…" - Participant K

"The health coordination meeting helped in information sharing. There was a good level of coordination among us. Therefore, I believe that health coordination meeting on basis of coordination was very fruitful." - Participant G

"…I think it was more information sharing than coordination. In the beginning, it was okay, but later since its health cluster there required provincial level, other sub-clusters, it should have been more on coordination, policies, monitoring, I think we should have gone in that way, especially in the leading part, we should have more matured." - Participant F

Theme 2: Process

Participants informed that the information was shared from the grass-root level initially, however, the information and communication were much more limited and repetitive later. The participants related the limitation in information to the decreased information from sub-clusters and grass-root level.

> *"Yes, previously when cluster meeting started, provincial presentations were done, they advocated on the activities done in the districts and gaps assessment, but then the meeting went about 2 hours long, and so provincial health coordination meetings was formed. This resulted meeting to end in short time, but that formed gaps in updating activities done at field level, issues faced during the time of intervention were updated in live, which later diminished."* - Participant J

> *"Lesson learnt shared during that time, in local level rather than thematic, practical way out was practiced. Activities carried out were addressed in the meetings before, but later it decreased, and I think we couldn't capture the information well."* - Participant G

> *"…certain monitoring of the sub clusters (Reproductive and Mental Health) could have been done because we lacked linkage and so whatever the sub clusters presented, we believed that to be right enough. Next, we should remember that we have fulfilled structure in the federal level, we fully utilized the district health office sources, during the earthquake and 2017 flood, the districts coordinated well during the time, later, the structure was also new and not all the provinces coordinated effectively."* - Participant C

> *…we missed service delivery point from basic grassroot level like palikas, health coordination unit etc. So, what is the linkage point from them, either its PHEOCs or any other source, that kind of information flow was missing."* - Participant K

"Next, in cluster coordination, it is not important for the same person or same division to share the information, of course, that's important but I think we should move much beyond that especially in a long row like 72 coordination meetings... The decrease in number of participants can indicate that our content and delivery was not appropriate enough. I felt like demand side from many local levels were missed, beneficiaries were never discussed…" - Participant F

There were mixed responses on addressing issues and queries from the health coordination meetings. However, one of the participants admired the documentation of the meetings (meeting notes and documents).

"…the coordination meetings held before was enthusiastic and issues were addressed but during the second phase, our issues were not equally addressed, even in provincial level, they started shifting work to one another." - Participant L

"Also, usually meetings are one-sided but in health coordination meeting, questions in chat box were also addressed. This might seem minor, but in my point of view following the same trend over 72 health coordination meetings, this a very good initiative taken…The way that meeting notes are formalized with action points, this is very important in coordination. Likewise, this built confidence in us as well. Reference documents, guidelines are shared time and again during and after the meeting."- Participant F

Theme 3: Participation

One of the participants highlighted the need for the Terms of Reference (ToR) for all the stakeholders in the health cluster coordination mechanism.

"During the beginning, health coordination was activated in a different manner from HEOC, ToR was not clear, some people even put forward their questions, another in logistics part, the information were confusing at times. Especially, during the staff turnover, many of us were unknown about our responsibility." - Participant F

Sending of the link to the organization only and the conflicting schedules of the participants caused the missing of the meetings by some participants.

"Our participation was frequent most of the times, but then there was constraint in sending meeting link…But some of them were missed due to conflicting schedules." - Participant L

Theme 4: Next steps

Participants were univocal on continuation of coordination meetings for preparedness, readiness, and response for disasters and other public health emergencies.

"Similarly, health coordination should also focus not only on disaster, but also in post-disaster, like the COVID cases have been dropping, we can also focus on discussing the other important areas of health. Since monsoon is approaching, we can also decide if we can take up the issue in health coordination or start preparing now. Also, I think regular coordination needs to be continued…. I feel like we need to bring out updated health coordination partners in a loop and update in resource mapping. We should develop a resource sharing platform and similarly, health coordination meetings can be regularized monthly. In general, health coordination mechanism needs to be in place." - Participant G

"…Hence, this meeting should be regularized. Likewise, since monsoon is following, we need to get prepared for the monsoon. Health coordination can look after the WASH and provincial clusters and update information…" - Participant C

"We can regularize health coordination and certain dedicated human resources should be in place. Nepal being challenged due to many aspects like climate, geography, etc., we cannot predict what comes in the way, information sharing mechanisms can be updated like via Viber group or any kind of emergency group." - Participant F

One of the participants also highlighted the role of health coordination meetings as the platform for progress monitoring and sharing.

"…but another important work is to monitor, just to see what progress has been made and share the documents with all in a continuous process. It is also one of the critical roles of health coordination division, since it builds up the confidence for all the partners who actually are keen to work." - Participant F

Suggestions/Recommendations

One of the participants suggested sharing of experiences/expert views in the meetings and the connection with the provincial and local level to be maintained in the meeting.

"There are a lot of places where we don't know exist, we need to seek expertise in those areas, the issues need to be addressed and existing guidelines should be easily accessible. The lessons learnt from our own experience need to be systematized in the upcoming days." - Participant E

"Because federal ministry of health is to be responsible to coordinate in health emergency as being driven by constitution, so I think that message must be set up and due to work of, for example, health emergency response in the province and the local government are playing their part in health emergency plan or response plan. They should sanction coordination; they are willing to coordinate with ministry and related any support which is of ministry of health and ultimately health coordination cluster... Also, I think the

connection between provincial and federal level should not be lost. We are discussing over here, but ground realities need to be addressed and solved, we need to consider those as well." - Participant E

Participants also requested to develop and/or revise the Terms of Reference (ToR) for Health Cluster Coordination Meetings.

"Therefore, I think the ToR (Terms of Reference) of the health cluster must be reviewed in all federal, provincial and district level because we lack in the process of information flow system and the way forward can be reviewing of ToR and handover system should be improved so that we're on the same page now onwards. Even though the sub-clusters are not formed, we should keep going with the health cluster…. One of the action points could be that we can include ToR from HEOC, and all the participants need to follow that. Agency's responsibility needs to be clear…. I think a revised ToR is a must. We should scale up the cluster activation like, during the normal period, we can get going with the preparedness while during emergency we get the activity done." - Participant C

"We need to identify the health partners and seek their own roles and responsibility in close coordination. Some relevant questions should be documented and addressed during or after the meeting." - Participant G

"During the beginning of cluster meeting, there were two kinds of institutions: 1) existing health institutions and 2) other non-health institutions that diverted their funds to work during COVID. Hence, we need to define their roles and update members so that they can engage beside COVID in later days…. Likewise, we do have a defined ToR for all the leads, co-leads, members etc. in our organization, maybe similar kind of defined ToR needs to be developed which might enhance effective participation further." - Participant J

Participants requested for one door mechanism for support and information sharing, through the health coordination meeting.

> *"But in context of coordination, two things seemed lacking, not only from center, but the entire system. Example, the needs/asks coming from different divisions, centers, was quite confusing. Rather than coming from multiple channels, it would have been quite easy if it carried one institutional value."* - Participant F

> *"…it should be improvised one door mechanism; there are different clusters, we have WASH cluster, and so many of the information were overlapping at times."* - Participant G

> *"…we created parallel system or multiple system, then there was struggle to share the information as quickly as possible. That was good. But I think we should have mainstreamed those multiple information systems, that created more confusion."* - Participant F

Participants suggested a mixed model for meetings with identification of focal person from each organization to reduce novelty of the meetings.

> *"Hence, we can switch on to mixed module so that the issues, gaps, and challenges faced in field level can also be reached directly to federal level. Along with this, the meeting time can also be maintained, and it might be more effective that way…. Even when we observe in meeting, there are almost 30/40 organizations, that might not be able to physically attend meetings during other emergencies, hence, we can also opt for mixed model, i.e., virtual and physical so that there's limited physical presence."* - Participant J

"Periodic sharing of template and updating it needs to be done and a focal person and alternative focal person needs to be allocated for information sharing to minimize gap. These things need to be mandated." - Participant C

"I've regularly attended the HC meetings, as there are focal points in relevant clusters, cluster leads/co-leads/ any focal point need to be present. Because, we are discussing on the same topic, but not reaching conclusion." - Participant J

"Also, individual participation would be crowded, but allocating focal person would be much helpful." - Participant L

"We should have a defined resource and a limit to our responsibilities. Maybe, we can allocate a communication person, it is very important to have a dedicated person for information." - Participant F

Participants also suggested developing a website for information sharing from and to the health cluster members.

"Likewise, we can opt for some innovative ideas to address the issues, this might not require a long report but rather it'll be issue based, we can create a website for that, it'll more be like learning by doing." - Participant E

"It'll be better if we could develop a website for sharing that information. Forming an interactive dashboard and linkage of all partners could be effective for future reference as well." - Participant F

Discussion

Ministry of Health and Population activated the health coordination meetings based on the cluster approach as defined in the National Disaster Response Framework (2013) for COVID-19 response early and conducted coordination meetings regularly to share information, identify

gaps, and streamline efforts. Partners appreciated the leadership of MoHP to initiate these coordination meetings early, fixing the time for its conduction, and sharing information and ideas in the meeting. The health coordination meetings were understood only as information sharing platforms by some partners and not as a platform supporting coordinated response. Coordination meetings only conducted as a reaction to sudden disasters, rather than proactive meetings, have been identified as an area for improvement regarding coordination.[13] The meetings were more frequent during the initial phase of COVID-19 and during the surge of cases. It has been a common observation that almost all underdeveloped and developing countries prone to disasters respond to disasters in a reactive rather than in a proactive manner.[14]

The meetings were documented with minutes shared to all the participants, which were praised by the health partners. However, it was found that in most meetings, follow-up through completion of action points were lacking and required improvement. As time went by, issues of repetition and monotonous content were observed, thus the suggestion to keep the meeting targeted to a specific objective and content relevant to the evolving situation. Coordination requires information scaled to different levels of responsibility and action, and should be updated or revalidated as per the changing situations.[15] There were observations of limited information captured from the sub-clusters and ground level, yet if included this considerably increase the overall meeting duration. A balance must be sought to keep the meeting manageable and productive while noting that the lack of involvement of sub-national level is perceived as poor coordination, unclear lines of authority, and contributes to communication breakdown.[16] This may be addressed, in part, by having clear terms of reference and roles and responsibilities. Conflicting schedules and sometimes the unavailability of meeting details to the focal person were mentioned as some of the reasons for absence from meetings. At the same time, it was observed that having a fixed meeting schedule per week was helpful for participants.

Meetings with management issues can be labelled as a "bad" meeting,[17] and thus require prior preparations and checks.

Using the health coordination meetings for sharing of experiences and expert views, involving and reaching out to the provincial and local level, and one door mechanism for support and information sharing were some of the suggestions from partners. Terms of Reference for all the stakeholders in the health coordination mechanism were highlighted as the major need to ensure active participation. Development of the Terms of Reference (ToR) and a hybrid model (virtual and in-person) for meeting with identification of focal person from each organization were also suggested. Continuation of health partner coordination following the cluster approach for disasters, epidemics, and other public health emergencies and its use for progress monitoring and sharing was identified as way forward. Structural aspects of coordination bodies including availability of coordination structures and regular meeting fora; clear roles, mandates and sufficient authority; the value of building on existing coordination mechanisms; and ongoing functioning of coordination bodies, before and after crises are identified to be important for effective coordination.[18] Development of a website for information sharing from and to the health cluster members was also advised. A socio-technical decision support system has been identified to be a promising strategy for creating the workable balance between order and flexibility required for coordination in practice.[15]

Two major activities of the health partner coordination were considered good by the health partners, one was recognized to be unsatisfactory, and the rest as satisfactory. Planning after identification of the priorities based on response analysis and acting in coordinated manner can support more effective response to the needs of the affected population. The purpose of the health cluster approach is to harmonize efforts and use available resources efficiently within the

framework of agreed objectives, priorities and strategies, for the benefit of the affected population(s) and towards collective outcome.[19]

Triangulation of information is the main strength of this study that supported to gain a deeper and broader understanding of the health partner coordination. The main limitation was retrospective data capturing which limited the scope of the data gathering based on an analytical framework.

Conclusion

COVID-19 has highlighted the importance of effective coordination for response to pandemic. Nepal effectively faced the challenge of coordinated response to COVID-19 through early initiation of health coordination meetings and smooth functioning of the health coordination mechanism and processes. There were several recommendations like continuation of coordination meeting, conducting meetings in a hybrid model (virtual and in-person), development of ToR, regular progress monitoring and sharing, and development of website for information sharing to further strengthen the health coordination throughout all the phases of disasters of which the most highlighted was the need on clarification of roles and responsibilities among humanitarian health partners made through the formulation of a specific terms of reference for humanitarian health partner coordination.

Acknowledgement

We would like to express our sincere gratitude to Dr. Roshan Pokharel, the secretary of the Ministry of Health and Population, and Dr. Rajesh Sambhajirao Pandav, Country Representative for WHO Nepal for their overall leadership and support in developing this research manuscript, including in the organization of a manuscript writing workshop.

Funding Statement

This research received no external funding.

Contributors

SKM, SKA, AG, and GD conceptualized the study; SKA, AG, and GD worked on the methodology; SKA, PKS, SN, GD, and BT contributed to document review, data verification, data curation, formal analysis and data interpretation; GD and BT contributed to preparation of first draft of the manuscript; SKM, SKA, and AG supervised throughout all the processes. All authors contributed to subsequent revisions and approved the final version submitted for publication.

Declaration of Interest

The authors declare no conflict of interest.

Disclaimer

The work represents the opinion of the authors and not that of the organization for whom they work.

References

1. Ministry of Health and Population. Responding to COVID-19: Health Sector Preparedness, Response and Lessons Learnt. Ministry of Health and Population. 2021. Weblink
2. World Health Organization. Health Cluster Coordination Guidance for Heads of WHO Country Offices as Cluster Lead Agency. 2019. Weblink
3. World Health Organization. The International Health Regulations (2005)- Third Edition. 2016. Weblink
4. Castañer X, Oliveira N. Collaboration, Coordination, and Cooperation Among Organizations: Establishing the Distinctive Meanings of These Terms Through a Systematic Literature Review. J Manage. 2020;46(6):965–1001. DOI

5. Bahadori M, Khankeh HR, Zaboli R, Raiszadeh M, Malmir I. Health Sector Coordination in Disasters: Barriers & Facilitators. Heal Emergencies Disasters Q. 2016;1(4):177–86. DOI
6. Shoaf K. Organizing the Health Sector for Response to Disasters. Cien Saude Colet. 2014;19(9):3705–15. DOI
7. The Global Cluster Coordination Group. Cluster Coordination Performance Monitoring: Guidance Note. 2014. Weblink
8. World Health Organization. WHO COVID-19 Strategic Preparedness and Response Plan: Operational Planning Guideline. 2021. Weblink
9. The Government of Nepal Ministry of Home Affairs. National Disaster Response Framework. 2013. Weblink
10. Student Activities and Leadership Center. Meeting Evaluation Checklist. Weblink
11. Magazine i.t.Toolkit. Post Meeting Assessment Steps. Weblink
12. Global Health Cluster. Annual CCPM Report 2020. 2021. Weblink
13. Zoraster RM. Barriers to Disaster Coordination: Health Sector Coordination in Banda Aceh Following The South Asia Tsunami. Prehosp Disaster Med. 2006;21(SUPPL.1). DOI
14. Izadkhah YO, Hosseini M. Using Proactive Means In Reducing Vulnerability To Natural Disasters. 14th World Conf Earthq Eng. 2008. Weblink
15. Comfort LK, Dunn M, Johnson D, Skertich R, Zagorecki A. Coordination In Complex Systems: Increasing Efficiency In Disaster Mitigation and Response. Int J Emerg Manag. 2004;2(1–2):62–80. DOI
16. Ngoy N, Oyugi B, Ouma PO, Conteh IN, Woldetsadik SF, Nanyunja M, et al. Coordination Mechanisms for COVID-19 In the WHO Regional Office for Africa. BMC Health Serv Res. 2022;22(1):1–17. DOI
17. Karl KA, Peluchette JV, Aghakhani N. Virtual Work Meetings During the COVID-19

Pandemic: The Good, Bad, and Ugly. Small Gr Res. 2022;53(3):343–65. DOI

18. Gooding K, Bertone MP, Loffreda G, Witter S. How can we strengthen partnership and coordination for health system emergency preparedness and response? Findings from a synthesis of experience across countries facing shocks. BMC Health Serv Res. 2022;22(1):1–19. DOI

19. World Health Organization. Health Cluster Guide: A Practical Handbook. 2020. Weblink

Supporting Information

S1 File: Supporting information1_Health partner coordination. Individual meeting's documents review tool

S2 File: Supporting information2_Health partner coordination. Tool for Assessment of Health Coordination established for COVID-19 Pandemic in Nepal

S3 File: Supporting information3_Health partner coordination. Interview Guide for Focus Group Discussion

Click here to access/download

Supporting Information - Compressed/ZIP File Archive

Supporting information.zip
